# New Treatment of Burns

**Published:** 1874-08

**Authors:** M. Olmsted Baldwin

**Affiliations:** Wamego, Kansas


ARTICLE VIII.—*A New Treatment for Burns.* By M. OLMSTED BALDWIN, M.D., Wamego, Kansas.

Without offering comments upon the many remedies now in use for burns, the writer ventures to present one which may perhaps in part be new, and not without advantages.

For a considerable time, it has been our custom to use eggs, the white and yolk together, well beaten up, as a local application, and the remedy has given a great degree of satisfaction. Our manner of applying, is, after the eggs are well beaten, to saturate old and well worn pieces of muslin therein, and spread over the injured surface, two or three layers thereof being superimposed; the relief is immediate and complete. The dressing should be renewed each twelve hours, meantime, should it become dry, it may be moistened by dripping water over it. After the first two or three dressings we add a little carbolic acid and glycerine, to correct any disagreeable odor, and also stimulate the healing process. The dressing is easily removed, and leaves a clean, fresh-looking surface, not attainable under the old processes.

